# Anatomy and Ultrastructural Details of the Compound Eyes of the Pear Psyllid, *Cacopsylla chinensis* (Yang et Li) (Hemiptera: Psyllidae)

**DOI:** 10.3390/insects17030287

**Published:** 2026-03-06

**Authors:** Hongfan Ran, Min Li, Yiping Niu, Aihong Ma, Xiaofan Yang

**Affiliations:** IPM Innovation Center of Hebei Province, Plant Protection Institute, Hebei Academy of Agriculture and Forestry Sciences, Baoding 071000, China; ranhongfan@163.com (H.R.); liminhappy-2003@163.com (M.L.); niu_20040118@126.com (Y.N.)

**Keywords:** Psyllidae, compound eye, ultrastructure, rhabdom, TEM

## Abstract

The compound eye of the family Psyllidae (Hemiptera: Sternorrhyncha) remains one of the least studied among hemipteran insects. Among the approximately 4000 psyllid species worldwide, the pear psyllid *Cacopsylla chinensis* (Yang et Li) is a major pest of pear trees in China. The ultrastructure of the compound eye of adult *C. chinensis* was investigated using a transmission electron microscope (n = 12 adult). The eyes are of the apposition type, lacking a clear zone. Each ommatidium features a laminated corneal lens (about 16.9 ± 1.7 μm in diameter and 7.6 ± 1.7 μm in thickness), a eucone crystalline cone, a centrally fused rhabdom formed by eight retinula cells (R1–R8), and both primary and secondary pigment cells. The 57.0 ± 3.6 μm long rhabdom exhibits a two-tiered structure: a distal region comprising R1-R7 and a proximal region composed of R1-R6 and R8, with R7 withdrawing. Throughout this structure, the rhabdomeres of R1-R6 contribute continuously along the entire length, while R7 and R8 are restricted to the distal and proximal regions, respectively. The microvilli of the rhabdom are arranged in two orthogonal directions. Based on the similarity between the compound eye of *C. chinensis* and those of other diurnal hemipterans, the evolution and function of eyes are briefly discussed.

## Simple Summary

The pear psyllid *Cacopsylla chinensis* (Yang et Li) is an oligophagous pest of pear trees in China, causing significant economic losses through both direct feeding and indirect sooty mold damage. In this study, we examined the detailed structure of the compound eye to evaluate its visual acuity. Using a transmission electron microscope, we found that the psyllid possesses apposition eyes, consisting of a plano-convex cornea, a crystalline cone, eight retinula cells forming a fused rhabdom, and both primary and secondary pigment cells. Interestingly, the rhabdom exhibits a distal region formed by R1–R7 and a proximal region including R1–R6 and R8. Understanding the ultrastructural morphology of compound eyes in *C. chinensis* is essential in understanding its visual capacity and host-seeking behavior, which will inform the development of effective pest management strategies.

## 1. Introduction

Compound eyes are the main visual organ in most insects, playing an important role in detecting motion, perceiving light intensity, and distinguishing spectral cues, thereby facilitating key processes such as survival, navigation, rhythm regulation, and reproduction [1,2,3]. These eyes consist of many structural and functional units called ommatidia, each typically comprising a corneal lens, a crystalline cone, a number of primary and secondary pigment cells, and a light-sensitive rhabdom formed by retinular cells [4,5]. Despite their structural uniformity, compound eyes exhibit diverse optical strategies and morphological modifications, reflecting adaptations to varying environmental conditions and ecological demands [6,7].

Compound eyes of insects are usually categorized into two basic types: apposition eye and superposition eye [1]. In the Hemiptera, most species possess apposition eyes with the major characteristic of lacking a clear zone [3,8]. Abundant information on the ultrastructural morphology of compound eyes in Hemiptera is available for heteropteran species, including Anthocoridae, Pentatomidae, and Reduviidae [8,9,10,11,12], as well as for aucheorrhynchan groups such as Cercopoidea, Cicadidae, and Ricanidae [13,14,15,16,17,18,19,20]. By contrast, a few studies have described the external morphology and internal structures of the compound eyes in Sternorrhyncha using light, transmission electron (TEM), and scanning electron (SEM) microscopes [21,22,23]. However, considerable structural variation exists across families, genera, and species, particularly in the organization of the rhabdomere system, which may form either a centrally fused or an open rhabdom, with corresponding differences in the arrangement of retinula cells [3,8,12,24]. Psyllidae, a family within Hemiptera: Sternorrhyncha, comprises approximately 4000 phytophagous species worldwide that are often highly host-specific [25,26]. However, the ultrastructure of psyllid compound eyes remains poorly characterized; detailed descriptions exist for only two species, i.e., *Glycaspis brimblecombei* (Moore) [27] and *Diaphorina citri* (Kuwayama) [28]. Among the 24 psyllid species that infest pear trees, *Cacopsylla chinensis* (Yang et Li) is considered the most harmful in China, causing substantial economic losses in the pear industry [29,30,31,32]. This phloem-sucking psylla damages pear trees both directly, through adults and nymphs feeding on young shoots and leaves, and indirectly, via the secretion of honeydew that causes severe sooty mold damage [33]. Given its small body size (2–3 mm) and limited active flight capacity, *C. chinensis* is largely restricted to the canopy of pear trees, where it faces a range of visually complex tasks to achieve feeding and reproduction. Therefore, detailed structural knowledge on the compound eyes in *C. chinensis* is essential in understanding the relationship between its visual adaptations and behavioral ecology within this specialized niche.

In this study, we investigate the morphology and ultrastructure of the compound eyes in the adult of *C. chinensis* using a transmission electron microscope, with a focus on the organization of ommatidia, photoreceptor cells, and screening pigments. This study is primarily descriptive, aiming to establish the fine structural basis of vision in this species. By comparing our findings with those of other hemipterans, we briefly discuss the visual acuity and potential functions.

## 2. Materials and Methods

### 2.1. Insect Collection

Adult specimens of *C. chinensis* were collected from a pear orchard in Fanzhuang Town (38.78° N, 114.87° E), Zhao County, Hebei Province, China, in June 2023. Prior to fixation, twelve live adults (6 females and 6 males) were exposed to 1000 lx for 2 h. The light-adapted specimens were decapitated and fixed under the same light intensities. Light intensity was measured using a radiometer (IL1700, International Light Technologies, Peabody, MA, USA).

### 2.2. Transmission Electron Microscope (TEM)

Following the procedure described by Yang et al. (2024) [34], the specimens were decapitated and immediately fixed in a mixture of 2.5% glutaraldehyde and 2.0% paraformaldehyde in phosphate-buffered saline (PBS, 0.1 M, pH 7.4) at 4 °C for 24 h. After fixation, the heads were washed in PBS and post-fixed in 1% osmium tetroxide (OsO_4_) in PBS at 4 °C for 2 h. The samples were then washed three times (10 min each) in deionized distilled water and dehydrated using a graded ethanol series (30%, 50%, 70%, 80%, and 90% for 10 min each and 100% for 30 min twice). Subsequently, the samples were infiltrated with acetone/Epon mixtures (3:1, 1:1, and 1:3) and then pure Epon. Finally, the samples were embedded in pure Epon 812 and polymerized at 45 °C for 24 h and 60 °C for 48 h.

For TEM observations, ultra-thin sections of 70 nm thickness were cut using a diamond knife on a Leica EM UC6 ultramicrotome (Leica, Nussloch, Germany). After double-staining with 2% uranyl acetate and 0.5% lead citrate, the sections were examined under an FEI Tecnai Spirit transmission electron microscope (FEI, Hillsboro, OR, USA) operated at 120 kV.

### 2.3. Morphometric Analyses

All histological measurements were analyzed using the Fiji software (v2.3.0; Fiji Is Just ImageJ) based on ImageJ2 (Rasband, W.S., National Institutes of Health, Bethesda, MD, USA). Longitudinal sections for TEM were used to measure the ommatidial lengths, rhabdom lengths, number of chitin layers, corneal thicknesses, corneal facets, and cone lengths. Distal and proximal rhabdom diameters and the diameters of pigment granulesin retinula cells were gathered from TEM transverse sections. The independent samples *t*-test was used to examine the difference between sexes. All statistical analyses were performed using IBM-SPSS v.27.0 (IBM, Armonk, NY, USA).

## 3. Results

**Figure 1:**
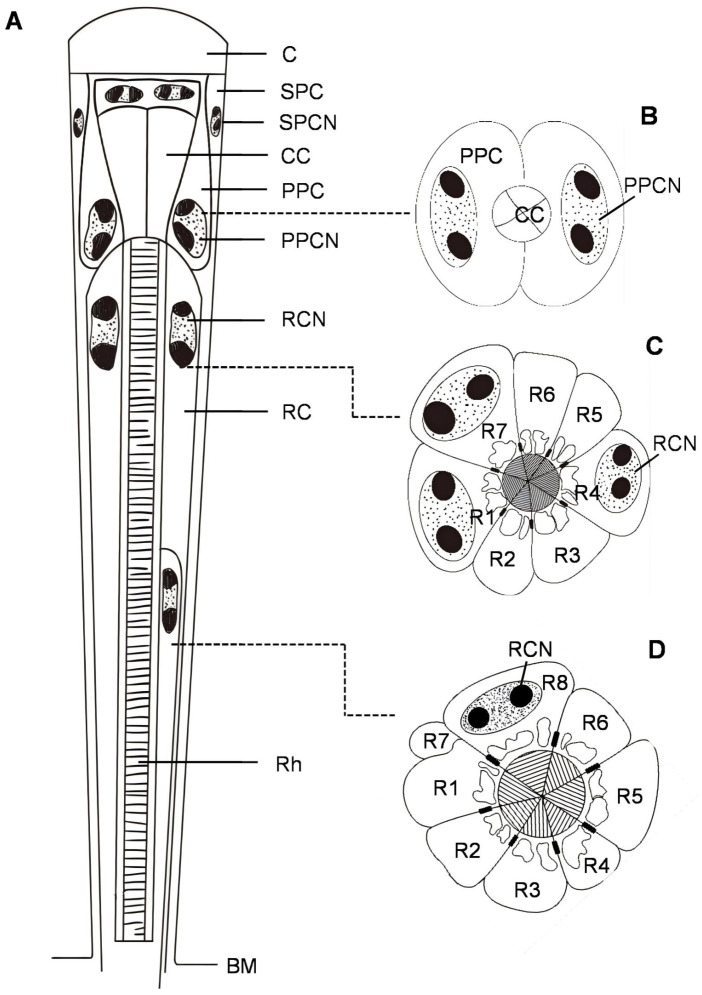
Semi-schematic drawings of the ommatidium of *C. chinensis*. (**A**) Longitudinal section of an ommatidium. (**B**) Transverse sections of the proximal region of the crystalline cone. The nuclei of primary pigment cells are in the proximal part of the cells. (**C**) Transverse sections of the distal region of the rhabdom, showing that seven retinula cells (R1–R7) contribute their rhabdomeres to the rhabdom. (**D**) Transverse sections of the proximal region of the rhabdom. The rhabdom consists of the rhabdomeres of six retinula cells (R1–R6) and the eighth retinula cell (R8), whereas R7 shifts toward the periphery and eventually ceases to contribute its rhabdomere to the rhabdom. BM, basal matrix; C, cornea; CC, crystalline cone; PPC, primary pigment cell; PPCN, primary pigment cell nucleus; RC, retinula cell; RCN, retinula cell nucleus; SPC, secondary pigment cell; SPCN, secondary pigment cell nucleus; Rh, rhabdom.

In *C. chinensis*, the ommatidia of the compound eye are of the apposition type. Each ommatidium consists of two distinct structures: the dioptric apparatus (a corneal lens and a crystalline cone), surrounded by primary and secondary pigment cells, and the photoreceptive layer. In the photoreceptive layer, eight retinula cells (R1–R8) form a fused rhabdom, which is distally connected to the crystalline cone. Semi-schematic diagrams of the ommatidium in *C. chinensis* are shown in Figure 1. Electron microscopic observations of the central eye region revealed no sexual dimorphism; thus, the measurements from males and females were combined (Table 1; see Table S1 for separate values). The total length of the ommatidium is on average 76.0 ± 3.0 μm, while the average interommatidial angle is 1.9 ± 0.6 deg, determined from the diameter of the corneal lens (15.6 ± 0.6 μm) and the eye radius of 87.8 ± 5.2 μm.

### 3.1. Dioptric Apparatus

**Figure 2:**
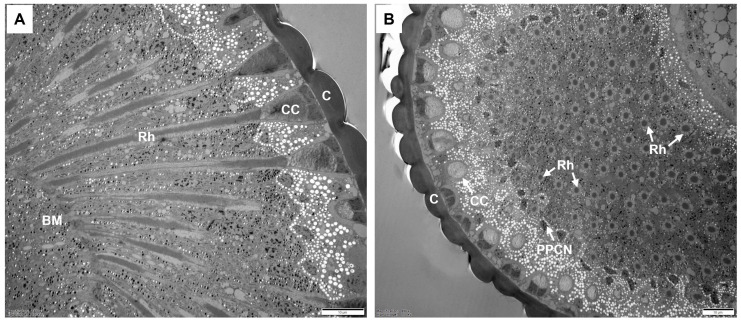
TEM micrographs of the ommatidial organization of the compound eyes in adult *C. chinensis.* (**A**) Longitudinal section of the ommatidia showing the corneal lens, crystalline cone, rhabdom layer, and basal matrix. (**B**) Transverse sections through the ommatidia. BM, basal matrix; C, cornea; CC, crystalline cone; PPCN, primary pigment cell nucleus; Rh, rhabdom. Scale bar: (**A**,**B**) = 10 μm.

**Figure 3:**
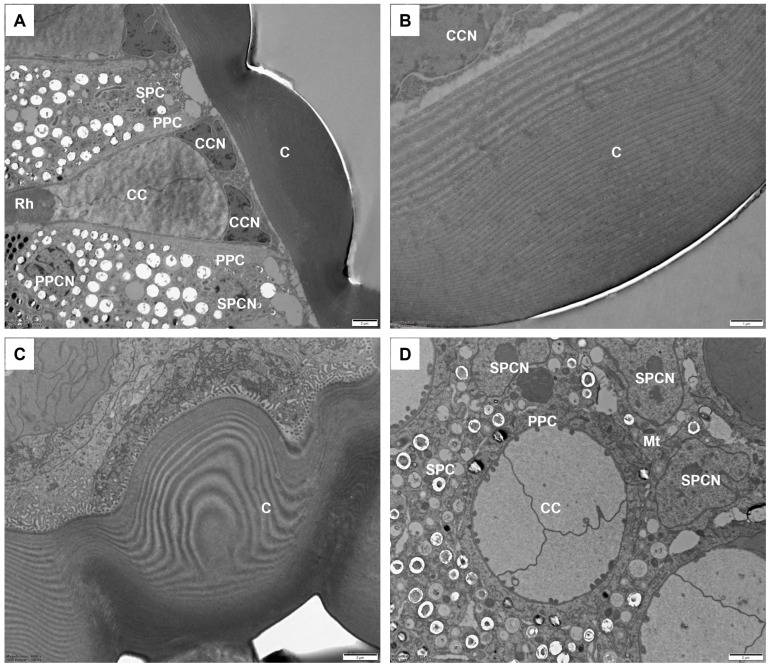
TEM micrographs of the distal part of the ommatidia in adult *C. chinensis*. (**A**) Longitudinal section of the dioptric apparatus, showing that the crystalline cone is surrounded by two primary pigment cells with large nuclei. (**B**) Longitudinal section of the laminated cornea. (**C**) Transverse section of the laminated cornea. (**D**) Transverse section of the distal region of the crystalline cone, showing the nuclei of secondary pigment cells. C, cornea; CC, crystalline cone; CCN, cone cell nucleus; Mt, mitochondrion; PPC, primary pigment cell; PPCN, primary pigment cell nucleus; Rh, rhabdom; SPC, secondary pigment cell; SPCN, secondary pigment cell nucleus. Scale bar: (**A**,**C**,**D**) = 2 μm; (**B**) = 1 μm.

The cornea is a plano-convex lens, featuring a highly convex outer surface and a nearly flat inner surface (Figure 2 and Figure 3A). Its outer radius of curvature is approximately 11.5 ± 1.0 μm. The cornea is about 16.9 ± 1.7 μm in diameter, with a maximum thickness of 7.6 ± 1.7 μm at the center of each facet. The cornea is laminated with approximately 40 ± 3 chitin layers, which are loosely arranged in the distal region but become more densely packed toward the basal portion (Figure 3B,C).

Beneath the cornea is the crystalline cone, which is of the eucone type. It is formed by four cone cells and has an average length of 15.6 ± 0.6 μm. Each cone cell contains a large nucleus located in the distalmost region, but no other organelles were observed. In a longitudinal section, the crystalline cone exhibits a funnel-like shape, tapering from a distal diameter of 11.1 ± 0.6 μm to a proximal tip width comparable to that of the rhabdom (Figure 3A). The crystalline cone connects directly to the distal end of the rhabdom. Each crystalline cone cell projects a thin root from its proximal end (Figure 2A), running along the rhabdom down to the basal matrix.

### 3.2. Pigment Cells

**Figure 4:**
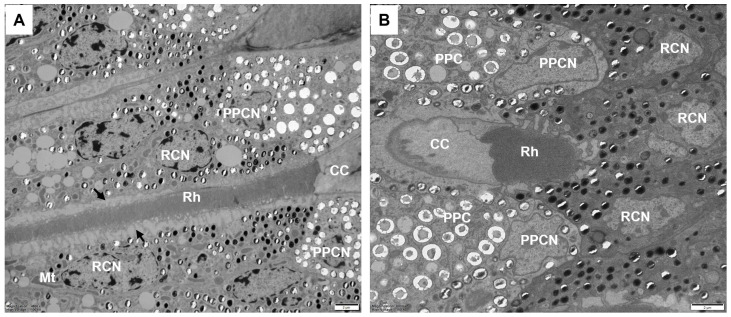
Junction of the crystalline cone and rhabdom. (**A**) Longitudinal section showing the primary pigment cells with large nuclei positioned below the distal tip of the fused rhabdom. Black arrows indicate endoplasmic cisternae around the periphery of the rhabdom. (**B**) Transverse section showing the four cone cells with primary pigment cells in contact with the fused rhabdom. CC, crystalline cone; Mt, mitochondrion; PPC, primary pigment cell; PPCN, primary pigment cell nucleus; RCN, retinular cell nucleus; Rh, rhabdom. Scale bar: (**A**,**B**) = 10 μm.

The two primary pigment cells envelop the crystalline cone from the distal to proximal ends, with their proximal regions in contact with the distal end of the retinula cells. Their cytoplasm contains numerous electron-lucent granules and few electron-dense pigment granules. A large nucleus almost occupies the entire proximal region of each primary pigment cell (Figure 3A and Figure 4A). An undetermined number of secondary pigment cells surround the primary pigment cells in each ommatidium (Figure 3D). These cells extend proximally to fill the space between adjacent ommatidia and contain numerous spherical electron-dense pigment granules. The nuclei of the secondary pigment cells are located in the distal regions, where their cytoplasm contains abundant mitochondria.

### 3.3. Retinula Cells and Rhabdom

**Figure 5:**
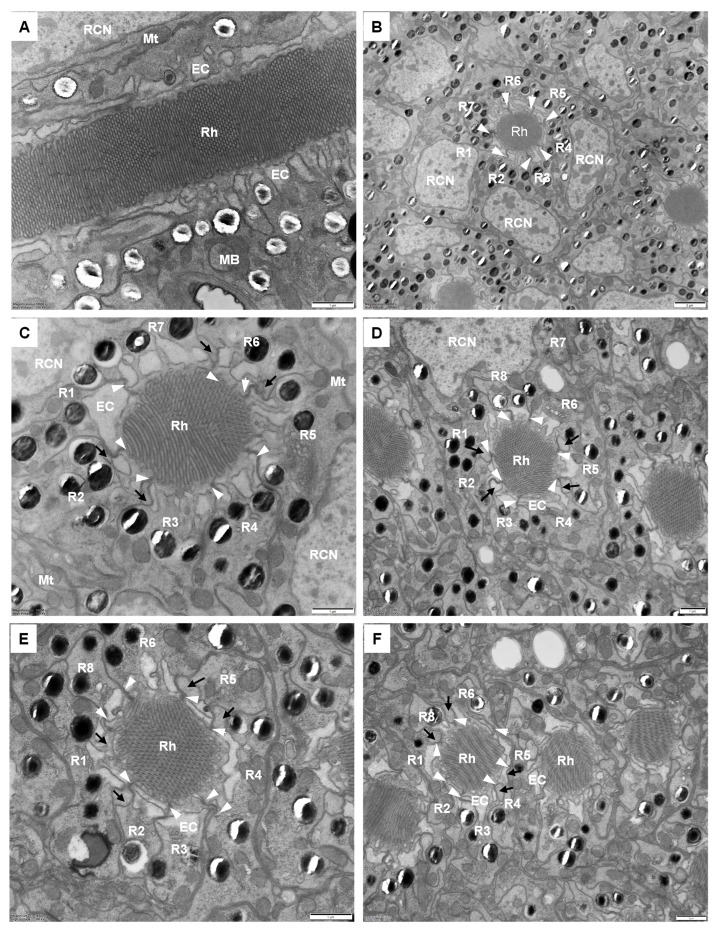
TEM micrographs of the retinula cells and rhabdom in adult *C. chinensis*. (**A**) Longitudinal section of the distal rhabdom, showing the ordered bands of microvilli. (**B**) Transverse section of the distal rhabdom, showing that seven retinula cells (R1–R7) contribute their rhabdomeres to the fused rhabdom. Endoplasmic cisternae (EC)-like vacuoles gather together and spread around the periphery of the rhabdom. (**C**) Transverse section with a higher magnification of the distal rhabdom. Note the desmosomes (white arrowheads) and four cone cell roots (black arrows) between each two adjacent retinula cells. (**D**) Transverse section of the proximal rhabdom formed by the rhabdomeres of R1-R6 and R8. The nucleus of the eighth retinula cell can be found in this region. (**E**) Area just below the nuclear region of the eighth retinula cells. (**F**) Transverse section through the most proximal rhabdom, located just above the basal matrix. EC, endoplasmic cisternae; MB, multivesicular body; Mt, mitochondrion; R1–R8, retinular cell; RCN, retinular cell nucleus; Rh, rhabdom. Scale bar: (**A**,**C**,**D**,**E**,**F**) = 1 μm; (**B**) = 2 μm.

Under the crystalline cone lie eight retinular cells forming a centrally fused rhabdom, measuring approximately 57.0 ± 3.6 μm in length. Retinular cells are numbered following the system by Friedrich et al. (2011) [35]. In all ommatidia, R1-R6 cells contribute rhabdomeres along their entire length, from the proximal tip of the crystalline cone to just above the basal matrix. In contrast, R7 and the proximally located R8 contribute exclusively to the distal and proximal regions of the rhabdom, respectively (Figure 5B–F). In the transverse section, the rhabdomeric microvilli of all retinula cells are arranged radially around the optical axis.

In the distal region, the R1–R7 cells contribute their rhabdomeres to form a distal rhabdom measuring 3.0 ± 0.2 μm in diameter (Figure 5B). Retinula cells are distally connected to primary pigment cells at the junction between the crystalline cone and the retinula (Figure 4). In longitudinal sections, the nuclei of R1–R7 cells appear as long, elliptic shapes and are all positioned within the same plane in the distal region of the retinula cells. The microvilli of the rhabdomeres are arranged in two orthogonal orientations, with microvilli within each orientation parallel to each other. This structural organization produces the banded appearance observed in longitudinal sections of the rhabdom (Figure 5A). Further proximally, the R8 cell becomes positioned between R6 and R7 cells and then extends proximally to contribute its rhabdomere to the proximal rhabdom, while R7 shifts peripherally and withdraws from rhabdom formation (Figure 5D–F). At this level, the rhabdom consists of rhabdomeres from R1-R6 and R8 cells, narrowing to about 2.4 ± 0.2 μm in diameter. The nucleus of the R8 cell is located near the middle of the rhabdom, occupying nearly the entire space of the cytoplasm. The retinula cells are connected by desmosomes, which are positioned adjacent to the cone cell root (Figure 5B–F). Their cytoplasm contains numerous spherical electron-dense pigment granules, approximately 0.54 ± 0.08 μm in diameter. Close to the rhabdom, endoplasmic cisternae merge into large electron-lucent palisades surrounding the rhabdom. In addition, other common organelles such as mitochondria, multivesicular bodies, and endoplasmic reticula are distributed in the retinula cells.

### 3.4. Basal Matrix

**Figure 6:**
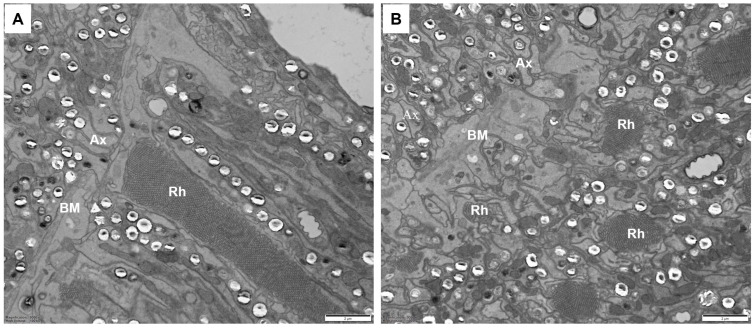
TEM micrographs of the basal matrix in adult *C. chinensis*. (**A**) Longitudinal section of the basal matrix, showing that axonal bundles penetrate the basal matrix through an obvious ostiole. (**B**) Transverse section of an axon bundle with eight fibers below the basal matrix. AX, axon; BM, basal matrix; Rh, rhabdom. Scale bar: (**A**,**B**) = 2 um.

The basal matrix between the retina and the lamina is about 0.41 ± 0.04 μm thick (Figure 6). Within each ommatidium, the eight retinula cells turn into axons, which gather into a bundle passing through a round perforation to the lamina. Numerous neurofilaments and mitochondria are distributed through the cytoplasm of these axons. In addition, large nuclei and electron-dense pigment granules are present below the basal matrix.

## 4. Discussion

This is the first study to provide a detailed ultrastructural description of the compound eyes in adult *C. chinensis* using a transmission electron microscope. The eyes of *C. chinensis* are of the apposition type and have a fused rhabdom, a feature also observed in other Sternorrhyncha families, including Aphididae [21,23], Aleyrodidae [22], and within Psylloidea for *D. citri* [28], as well as in diverse Auchenorrhynchan families, such as Cicadidae, Cicadellidae, and Cercopidae [13,15,16,17,18,19,20]. Fischer et al. (2000) noted that Sternorrhyncha and Auchenorrhyncha members typically possess a fused rhabdom, whereas Heteroptera species consistently exhibit an open one [8]. Thus, the consistent pattern of fused versus open rhabdoms may serve as a phylogenetically informative characteristic within Hemiptera.

In the ommatidia of *C. chinensis*, the proximal end of the crystalline cone is directly connected to the distal end of the rhabdom, similar to other hemipterans, such as *D. citri* (Psyllidae), *P. spumarius* (Cercopidae), and some species of Cicadidae and Cicadellidae, including *Meimuna mongolica*, *Psaltoda moerens*, *Empoasca vitis*, and *R. speculum* [13,16,17,18,20,28]. In contrast to this pattern, the rhabdom in *Montandoniola moraguesi* (Anthocoridae) is distally enveloped by the crystalline cone, while *C. versicolor* (Cercopidae) exhibits a reversed configuration with the rhabdom enveloping the crystalline cone [12,15]. These structural differences among Hemiptera species may represent important adaptive strategies and provide valuable morphological evidence for understanding their evolution, visual adaptation, and functional implications. Compared to other Sternorrhyncha species, *C. chinensis* possesses a larger rhabdom diameter, measuring 3.0 ± 0.2 μm distally [21,28]. Large rhabdom diameters are often associated with small insect body sizes, a known adaptation to compound eye miniaturization. For example, the parasitoid wasp *Megaphragma mymaripenne* (0.2 mm in body size) exhibits a rhabdom diameter of up to 2.4 μm, while those of *Trichogramma evanescens* (0.3–0.4 mm in body size) and *Anaphes flavipes* (0.45 mm in body size) measure approximately 1.7 μm and 1.4 μm, respectively [36]. Functionally, a large rhabdom diameter increases the total photon catch, and when exceeding about 2 μm, the rhabdom acts as a light guide, trapping light inside through total internal reflection [37].

Despite sharing eight typical retinula cells, the arrangement of these cells differs notably between Sternorrhyncha and Auchenorrhyncha. In *C. chinensis*, the rhabdomeres of R1–R6 contribute along nearly the entire rhabdom length, with R7 and R8 restricted to the distal and proximal regions, respectively. A different pattern is observed in *R. speculum*, where the distal rhabdom is formed only by R1-R6, with R7 and R8 interposed between them at more proximal levels [20]. In some other Hemiptera, such as *D. citri*, *C. versicolor*, *M. mongolica*, *P. spumarius*, and *P. moerens*, however, the eight retinula cells contribute their rhabdomeres more uniformly along the rhabdom length [13,15,16,18,28]. These organizational differences in retinula cells suggest distinct developmental pathways and functional specializations for visual adaptation among different hemipteran groups with a fused rhabdom. Further comparative studies will be essential in elucidating the functional significance of these structural variations. An orthogonal arrangement of microvilli can be found in the rhabdom of *C. chinensis*, a structural feature often associated with polarization sensitivity in insects. For comparison, *P. moerens* possesses a small, specialized dorsal rim area (DRA) dedicated to polarized light detection [16]. However, while behavioral and electrophysiological evidence for polarization vision is well established in insects such as locusts, ants, crickets, dung beetles, honeybees, and butterflies [38,39,40,41,42,43], conclusive experimental evidence within Hemiptera remains remarkably limited. In the absence of direct functional or specialized structural data (e.g., a distinct DRA) in *C. chinensis*, any inference regarding polarization sensitivity in this species remains speculative. The compound eyes in *C. chinensis* appear to reflect ecological adaptations to a diurnal lifestyle that facilitate high spatial resolution in bright light, thereby enabling it to perform visually complex tasks within the specific microhabitat of its host plant. Previous studies have shown that several related psyllid species can use visual cues to guide their host selection, with a particular preference for specific wavelengths that match the reflectance spectra of their host plants [44,45]. For example, *Anoeconesossa bundoorensis* and *Glycaspis brimblecombei* are strongly attracted to red wavelengths, while *Ctenarytaina eucalypti* and *Ctenarytaina bipartita* prefer yellow and green wavelengths [45]. Among these four psyllid species studied, *G. brimblecombei* has the largest ommatidial facets (approximately 20–30 μm in diameter) and the greatest visual acuity, characterized by an inter-ommatidial angle of 6.3 deg and an average resolution of 0.125 cyc/deg [27,46]. In apposition eyes, smaller interommatidial angles enhance spatial resolution [1,47]. Therefore, the smaller interommatidial angles (about 1.9 ± 0.6 deg) observed in *C. chinensis*, relative to *G. brimblecombei*, suggest a higher spatial resolution, which may be functionally correlated with its limited active flight capacity. In conclusion, the psyllid *C. chinensis* possesses a typical apposition eye with a fused rhabdom. This study provides a detailed ultrastructural description of the visual system of the Sternorrhyncha, particularly for the family Psyllidae. The structural features not only support the established phylogenetic distinction from Heteroptera but also offer an anatomical foundation for understanding the visual ecology of psyllids. Given that retinula cell arrangements and their visual pigments underpin spectral sensitivity in insects, the specializations observed here highlight the need for physiological research to explore potential spectral diversity.

## Figures and Tables

**Table 1:** Measurements on the compound eyes of *Cacopsylla chinensis*.

Structural Compositions	Morphological Data	N	Unit	Measurements
Ommatidium	Length	15	μm	76.0 ± 3.0
	Interommatidial angle	6	deg	1.9 ± 0.6
Cornea	Diameter	19	μm	16.9 ± 1.7
	Maximum thickness	17	μm	7.6 ± 1.7
	Number of chitin layers	10	-	40 ± 3
	Radius curvature of cornea	12	μm	11.5 ± 1.0
Crystalline cone	Length	12	μm	15.6 ± 0.6
	Diameter (distal)	15	μm	11.1 ± 0.6
Rhabdom	Length	14	μm	57.0 ± 3.6
	Diameter (distal)	24	μm	3.0 ± 0.2
	Diameter (proximal)	24	μm	2.4 ± 0.2
	Diameter of pigment granules	65	μm	0.54 ± 0.08
Basal matrix	Thickness	10	μm	0.41 ± 0.04

Data are presented as the mean ± SD. N indicates the sample size.

## Data Availability

The original contributions presented in this study are included in the article/Supplementary Material. Further inquiries can be directed to the corresponding authors.

## Supplementary Materials

The following supporting information can be downloaded at: https://www.mdpi.com/article/10.3390/insects17030287/s1. Table S1: Measured parameters of the compound eyes of *Cacopsylla chinensis.*
