# Supplementary material for: Anatomy and Ultrastructural Details of the Compound Eyes of the Pear Psyllid, Cacopsylla chinensis (Yang et Li) (Hemiptera: Psyllidae)
Source: Insects. 2026 Mar 6;17(3):287. doi: 10.3390/insects17030287 (PMC13027190; doi:10.3390/insects17030287)
Supplement: Supplementary file 1 [file insects-17-00287-s001.zip › insects-4172246-supplementary.pdf]

Table S1 Measured parameters of the compound eyes of *Cacopsylla chinensis*.

Parameter	Unit	Measurements (mean ± SD)				*P*
		N	Female	N	Male	
Ommatidia length	μm	7	75.51 ± 3.25	8	76.62 ± 2.78	0.524
Diameter of cornea	μm	10	16.87 ± 1.78	9	16.76 ± 1.63	0.763
Maximum thickness of cornea	μm	8	7.61 ± 0.76	9	7.50 ± 0.75	0.747
Number of chitin layers	-	5	40 ± 3	5	40 ± 2	0.909
Radius curvature cornea	μm	6	11.51 ± 0.84	6	11.54 ± 1.25	0.955
Conelength	μm	7	15.44 ± 0.59	6	15.73 ± 0.48	0.160
Conediameter (distal)	μm	8	11.22 ± 0.52	7	10.98 ± 0.66	0.445
Rhabdom length	μm	7	56.81 ± 3.86	7	57.09 ± 3.56	0.891
Rhabdom diameter (distal)	μm	12	3.02 ± 0.15	12	3.01 ± 0.18	0.800
Rhabdom diameter (proximal)	μm	12	2.38 ± 0.17	12	2.44 ± 0.18	0.455
Diameter of pigment granules	μm	32	0.53 ± 0.09	32	0.55 ± 0.07	0.368
Thickness of basal matrix	μm	5	0.41 ± 0.04	5	0.42 ± 0.04	0.633

Data are presented as the mean ± SD. N indicates the sample size. The datasets were tested for significant differences between the sexes ($P < 0.05$) by independent samples t-test.
